# Molecular genetic basis of epidermolysis bullosa

**DOI:** 10.18699/VJGB-23-04

**Published:** 2023-03

**Authors:** Yu.Yu. Kotalevskaya, V.A. Stepanov

**Affiliations:** Moscow Regional Research and Clinical Institute, Moscow, Russia Charitable Foundation “BELA. Butterfly Children”, Moscow, Russia; Research Institute of Medical Genetics, Tomsk National Research Medical Center of the Russian Academy of Sciences, Tomsk, Russia

**Keywords:** epidermolysis bullosa, pathogenesis, genotype-phenotype correlations, heterogeneity, буллезный эпидермолиз, патогенез, корреляции генотип-фенотип, гетерогенность

## Abstract

Epidermolysis bullosa (EB) is an inherited disorder of skin fragility, caused by mutations in a large number of genes associated with skin integrity and dermal-epidermal adhesion. Skin fragility is manifested by a decrease in resistance to external mechanical influences, the clinical signs of which are the formation of blisters, erosions and wounds on the skin and mucous membranes. EB is a multisystemic disease and characterized by a wide phenotypic spectrum with extracutaneous complications in severe types, besides the skin and mucous membranes, with high mortality. More than 30 clinical subtypes have been identified, which are grouped into four main types: simplex EB, junctional EB, dystrophic EB and Kindler syndrome. To date, pathogenic variants in 16 different genes are associated with EB and encode proteins that are part of the skin anchoring structures or are signaling proteins. Genetic mutations cause dysfunction of cellular structures, differentiation, proliferation and apoptosis of cells, leading to mechanical instability of the skin. The formation of reduced proteins or decrease in their level leads mainly to functional disorders, forming mild or intermediate severe phenotypes. Absent protein expression is a result of null genetic variants and leads to structural abnormalities, causing a severe clinical phenotype. For most of the genes involved in the pathogenesis of EB, certain relationships have been established between the type and position of genetic variant and the severity of the clinical manifestations of the disease. Establishing an accurate diagnosis depends on the correlation of clinical, genealogical and immunohistological data in combination with molecular genetic testing. In general, the study of clinical, genetic and ultrastructural changes in EB has significantly expanded the understanding of the natural history of the disease and supplemented the data on genotype-phenotype correlations, promotes the search and study of epigenetic and non-genetic disease modifier factors, and also allows developing approaches to radical treatment of the disease. New advances of sequencing technologies have made it possible to describe new phenotypes and study their genetic and molecular mechanisms. This article describes the pathogenetic aspects and genes that cause main and rare syndromic subtypes of EB.

## Abstract (ru)

Буллезный эпидермолиз (БЭ) – наследственное нарушение, вызывающее хрупкость кожи, обусловленную изменениями генов, отвечающих за целостность кожи и дермо-эпидермальную адгезию. Хрупкость кожи проявляется снижением устойчивости к внешним механическим воздействиям, клинические признаки которой – образование пузырей, эрозий и ран на коже и слизистых оболочках. Для БЭ характерен широкий фенотипический спектр, при тяжелых типах, кроме кожи и слизистых, отмечаются мультисистемность поражения и развитие внекожных осложнений, высокая летальность. Выделено более 30 клинических подтипов БЭ, сгруппированных в четыре основных типа: простой, пограничный, дистрофический БЭ и синдром Киндлера. На сегодняшний день БЭ обусловливают патогенные варианты в 16 различных генах, которые кодируют белки, входящие в состав крепящих структур кожи, и сигнальные белки. Генетические дефекты в этих генах служат причиной нарушения функции клеточных структур, процессов дифференцировки, пролиферации и апоптоза клеток, приводя к механической неустойчивости кожи. Образование укороченных белков или уменьшение их количества обуславливает в основном функциональные нарушения, формируя легкие или среднетяжелые фенотипы. При нулевых генетических вариантах, вследствие которых экспрессия белка утрачивается полностью, возникают структурные нарушения, влекущие тяжелую клиническую картину. Для большинства вовлеченных в патогенез БЭ генов обнаружены определенные связи между характером и локализацией генетических де- фектов с тяжестью клинических проявлений заболевания. Установление точного диагноза зависит от корре- ляции клинических, генеалогических и иммуногистологических данных в сочетании с молекулярно-генетиче- ским исследованием. В целом изучение клинических, генетических и ультраструктурных изменений при БЭ значительно расширяет понимание естественного течения заболевания и пополняет данные о корреляциях генотип-фенотип, способствует поиску и изучению эпигенетических и негенетических факторов-модификато- ров заболевания, а также разработке подходов к радикальному лечению заболевания. Новые возможности технологий секвенирования позволили описать новые фенотипы и изучить их генетические и молекулярные механизмы. В настоящей статье описаны патогенетические аспекты и гены, вызывающие классические и ред- кие синдромальные подтипы БЭ.

## Introduction

Epidermolysis bullosa (EB) is a group of rare and currently incurable genetically determined hereditary skin diseases. The disease is characterized by fragility of the skin and mucous membranes that occurs with mechanical trauma, seemingly insignificant in terms of shear force, often accompanied by damage to nails, teeth and hair (Pânzaru et al., 2022). The spectrum of characteristic skin manifestations is wide and includes blisters, erosions, wounds that can become chronic, scarring, crusting, milia, skin atrophy, and dyspigmentation. In rare subtypes, it is possible not only to damage the skin, but also muscles, the gastrointestinal tract, kidneys, etc., which is due to the nature of the expression of the defective protein.

The severity of the disease varies from phenotypically mild to severe disabling or lethal variants, which determines the expected prognosis of life expectancy. Severe EB subtypes develop as systemic diseases with secondary multiple organ damage and developmental delay, anemia, affect heart and bones, movement disorders, early susceptibility to skin cancer, and premature death. The treatment of EB is exclusively symptomatic and is aimed at the prevention of mechanical injuries, wound care, treatment of infectious complications and extracutaneous manifestations of the disease. To date, no therapeutic approaches have been able to cure EB patients (Pânzaru et al., 2022).

Epidermolysis bullosa is a demonstrative model of mechanobullous disease, and the study of the underlying mechanisms has made it possible to make significant progress in understanding the fundamentals of the physiology and pathophysiology of the skin. The gained knowledge about EB was reflected in the classification, which was revised several times over the past decade by an international consensus group (Has et al., 2020a). Epidermolysis bullosa is divided into four main types – simplex EB (EBS), junctional EB (JEB), dystrophic BE (DEB) and Kindler’s syndrome (KS), which is based on the ultrastructural changes and the level of blisters in the skin and reflects the consequences of genetic defects on the protein function. Epidermolysis bullosa is clinically and genetically very heterogeneous, inherited in an autosomal dominant (AD) or autosomal recessive (AR) pattern of inheritance (Has et al., 2020a). Advances in understanding the pathogenesis of EB contribute to the development of potentially effective protein, cell and gene therapies (Has et al., 2020b).

The epidermal basal layer, basement membrane zone (BMZ) and extracellular matrix are key subregions that take central place in the pathophysiology of EB (Uitto et al., 2017) and genetic changes disturb the structure or function of their proteins (Mariath et al., 2020a). Pathogenic variants in 16 different genes determine the genetic and allelic heterogeneity of EB and the grouping of four main types of EB, including more than 30 clinical subtypes. EB-associated genes encode intracellular, transmembrane or extracellular proteins, mainly structural components of the cytoskeleton (keratin 5 and 14), BMZ (α6β4 integrin, type XVII collagen, laminin-332, type VII collagen, α3 integrin alpha subunit, kindlin-1) or intercellular adhesion proteins (desmoplakin, plakophilin, placoglobin) (see the Table) (Has, Bruckner- Tuderman, 2014). Table presents the key processes of pathogenesis leading to a certain phenotype.

**Table 1.**
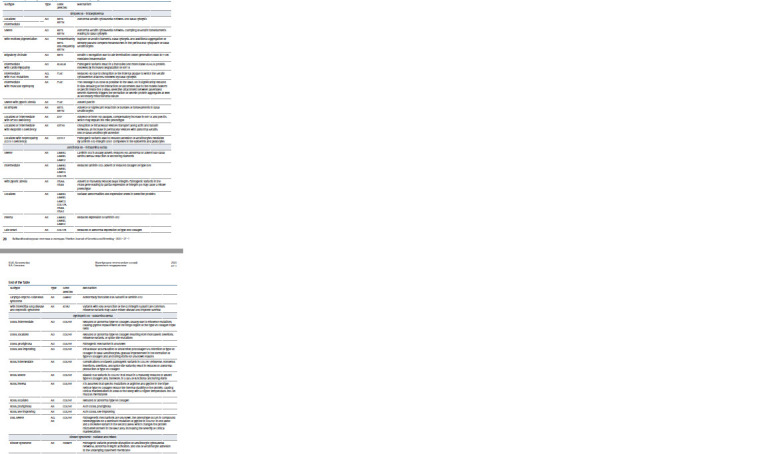
Classification of epidermolysis bullosa (EB) and main mechanisms of pathogenesis Notе. AD – autosomal dominant type of inheritance; AR – autosomal recessive type of inheritance; BMZ – basement membrane zone; HD – hemidesmosome; DDEB – dominant dystrophic epidermolysis bullosa; RDEB – recessive dystrophic epidermolysis bullosa.

## The main EB types

Simplex EB (EBS) is the most common type, accounting for about 70 % of all patients with EB (Has, Fischer, 2019), and includes 14 clinical subtypes according to the latest classification. Simplex EB has a wide range of severity, from mild with blistering of the palms and feet to generalized forms with extracutaneous lesions, sometimes fatal (Fine, 2010). Simplex EB is most often caused by defects in the keratin filaments of basal keratinocytes, has a different genetic basis: it is associated with changes in at least seven genes and represents the greatest clinical diversity.

Most subtypes of EBS are inherited in the AD pattern, although AR inheritance occurs in some regions of the world (Gostyńska et al., 2015; Vahidnezhad et al., 2019). The most common EBS subtypes observed in clinical practice are caused by mutations in the keratin 5 or 14 genes (70–80 % of cases), while according to the literature data, at least 17 % of patients with EBS had mutations de novo (Bolling et al., 2011; Wertheim-Tysarowska et al., 2016). In addition, EBS with AD inheritance may be associated with heterozygous variants in the PLEC or KLHL24 genes (Grilletta, 2019; Kiritsi et al., 2021). Rare digenic inheritance caused by mutations in the KRT5 and KRT14 genes have also been described in patients with EBS (Sathishkumar et al., 2016).

Keratin 5 and keratin 14 have a similar protein structure consisting of a central α-helical rod domain that is responsible for the polymerization of these proteins to form keratin tonofilaments. The core domain is subdivided into segments 1A, 1B, 2A and 2B by flexible linkers L1, L12 and L2, flanked by variable domains V1 and V2 in both proteins. Also, keratin 5 has a conserved H1 and H2 homology domain. The KRT5 and KRT14 genes are expressed in the basal keratinocytes of the epidermis, where their protein products combine to form heterodimeric molecules. The K5 and K14 dimers are the main components of the keratinocyte intermediate filament system, which assemble into an intracellular network (Bunick, Milstone, 2017).

Among the pathogenic variants in the KRT5 and KRT14 genes predominate dominant missense variants that affect the ability of keratins to interact with their partner. The locations of the pathogenic variant in the functional domains of the KRT5 or KRT14 genes are of key importance (Arin et al., 2010). Dominant-negative pathogenic variants are grouped at the beginning of 1A or the end of 2B segments of the helical rod domain of KRT5 and KRT14 and are typical of severe generalized EBS, because these domains are highly conserved and are considered critical for filament assembly.

The most common pathogenic variants are: p.Glu477Lys in the KRT5 gene and p.Arg125Cys, p.Arg125His, p.Asn123Ser in the KRT14 gene (Bolling et al., 2011; Vahidnezhad et al., 2016). In moderate EBS, pathogenic variants are located in the second part of segments 1A or 2B of the core domain of KRT5 and KRT14. In this subtype, they do not alter the process of keratin elongation during filament assembly, but impair their function (Has, Bruckner-Tuderman, 2014). In the localized EBS subtype, pathogenic variants are clustered in both KRT5 and KRT14, usually outside the highly conserved core domain boundary motifs, as well as in L12 linkers, in addition, in the KRT5 gene in the H1 domain, causing structural instability of the filaments (Bardhan et al., 2020). More distinct correlations with the genotype were found in the EBS subtype with spotted pigmentation, which is associated with pathogenic variants in the V1 domain of the KRT5 gene, so the p.Pro25Leu variant accounts for 90–95 % of mutations in this subtype (Arin et al., 2010).

Severe and moderate EBS with AR inheritance is associated with rare pathogenic biallelic variants in KRT14 and KRT5, which are found in consanguineous families (Vahidnezhad et al., 2016). Homozygous mutations in the KRT5 gene result in a severe phenotype, extracutaneous manifestations, and early mortality (Has et al., 2006).

The latest revision of the EB classification characterized rare syndromic EBS subtypes associated with mutations in the PLEC, KLHL24, DST, EXPH5, and CD151 genes (see the Table); we will consider them below.

The plectin protein encoded by the PLEC gene is a cytoskeletal protein that links the network of intermediate filaments to HD and thus acts as a mediator of the mechanical stability of keratinocytes in the skin (Natsuga, 2015). A large number of alternatively spliced first exons of the plectin gene form multiple protein isoforms and determine different expression in tissues, which ensures clinical diversity and leads to four rare EBS phenotypes

Pathogenic variants in the PLEC gene were mainly found in exons 31 and 32, loss-of-function variants leading to more severe phenotypes such as EBS with pyloric atresia (EBS-AP) and, as a result of null variants of the PLEC gene, EBS with muscular dystrophy (EBS-MD), where skeletal muscle fibers lose their structural integrity due to defects in desmin filaments (Natsuga, 2015). Moderate EBS with AR inheritance is caused by a specific homozygous nonsense mutation p.Arg16X in the first exon encoding the plectin 1a isoform, resulting in the absence of only this specific isoform (Gostyńska et al., 2015). Also, in exon 31 of the PLEC gene, a dominant amino acid substitution p.Arg2110Trp was described, which leads to a partial loss of protein function and causes HD fragmentation (Kiritsi et al., 2021), which is clinically manifested as moderate EBS.

The KLHL24 protein belongs to a family of highly conserved proteins with BTB/kelch domains; pathogenic variants in the KLHL24 gene lead to dysregulation of autoubiquitination and change the regulation of degradation of keratin 14 mo-lecules and cause its fragmentation (Dhanoa et al., 2013). In the EBS subtype caused by mutations in the KLHL24 gene, in all described cases, a heterozygous variant was observed in the start codon, the most common being c.1A-G with a dominant negative effect (Bardhan et al., 2020). Also, 85 % of patients with this subtype of EBS at a young age develop dilated cardiomyopathy caused by KLHL24-mediated degradation of desmin, the main protein of cardiomyocyte intermediate filaments (Grilletta, 2019).

Dystonin (BPAG1) is a member of the plakin protein family (Ganani et al., 2021). The DST gene encodes the epithelial BPAG1-e isoform, which is a structural component of internal HD plaques and consists of a helical-helical rod domain and flanking N- and C-termini. The N-terminus of the BPAG1-e protein is involved in its integration into HD and has binding sites for type XVII collagen and β4 integrin, while the C-terminus is the key point of attachment of keratin intermediate filaments (Kumar et al., 2015). Mutations in BPAG1-e have been shown to be associated with impaired adhesion of keratinocytes, increased cell migration with reduced expression of β4-integrins on the cell surface (Ganani et al., 2021). Clinically, it leads to a mild phenotype.

The exophilin-5 protein, a RAB27b GTPase effector protein encoded by the EXPH5 gene, is not a structural component of intermediate filaments, desmosomes, or PD. Although its role is not fully known, it is assumed that it contributes to the regulation of intracellular transport of vesicles, including the control of their formation and movement along the actin and tubulin networks, as well as the secretion of exosomes (Natsuga et al., 2010). Single families are described with homozygous variants in the EXPH5 gene, leading to a frameshift, as well as in combination with nonsense variants. Mild clinical manifestations have been described.

In the epidermis, the expression of the transmembrane protein CD151 is localized in HD, binding to α6β4 integrin and stabilizing its interaction with laminin-332, and plays a critical role in the formation of the НD complex. CD151 mediates cell adhesion and intracellular vesicular transport of integrins. In the kidneys, it forms complexes with α3β1 and α6β1 integrins and is required for the correct assembly of glomerular and tubular basement membranes (Margadant et al., 2010). A defect in the CD151 protein determines the clinical manifestations in individuals with CD151-associated EBS, including nephropathy with proteinuria (Karamatic Crew et al., 2004).

Junctional EB (JEB) is also a clinically and genetically heterogeneous group of skin fragility disorders, includes nine clinical subtypes, and is a rare type of EB (Has et al., 2020a). JEB subtypes have pathognomonic signs, for example, in severe generalized subtype, granulation tissue is rapidly formed in typical places, and mortality is high (Kiritsi et al., 2011). Phenotypic variability in JEB is extremely wide – from only nail dystrophy to death in the first year of life. Pathogenic variants in seven different genes lead to the development of JEB, all subtypes are inherited in the AR type. Pathogenic variants in the LAMA3, LAMB3, and LAMC2 genes encoding the α3, β3, and γ2 chains of laminin-332, as well as in the COL17A1 gene, encoding type XVII collagen, lead to the most common JEB subtypes (Uitto et al., 2016). Rare JEB phenotypes are associated with deficiency of a6β4 integrin, leading to the development of JEB with pyloric atresia and deficiency of the α3 subunit of a3β1 integrin, causing EBS with respiratory and renal involvement (Kiritsi et al., 2013).

The laminin-332 protein is a heterotrimer consisting of α3, β3, and γ2 chains, which are encoded by the LAMA3, LAMB3, and LAMC2 genes, respectively. Together with the extracellular domain of type XVII collagen, they form anchor filaments. The laminin-332 protein binds at its α-chain C-terminus to α3β1 integrins in focal adhesion sites and α6β4 integrins in HD, connecting the surface of basal keratinocytes to the dermal-epidermal BM (Dogic et al., 1998). In the dermis, the N-terminus of laminin-332 chains bind to type VII collagen, so that anchor filaments and anchor fibrils connect directly (Aumailley et al., 2003). Loss of laminin-332 expression causes extreme skin fragility and excess granulation tissue in generalized severe JEB. In laminin-332-deficient JEB subtypes, the LAMB3 gene is altered in 70 % of cases. Approximately 9 % of patients with JEB have mutations in the LAMA3 and LAMC2 genes, respectively (Varki et al., 2006; Uitto et al., 2016). The most common pathogenic variant is p.R635X, as a “hot” mutation point, which accounts for 45–63 % of all pathogenic alleles of the LAMB3 gene in generalized severe JEB, resulting in the absence of one of the three proteins that are assembled in laminin-332.

Mild manifestations of EB are caused by missense mutations, splicing site mutations, and deletions with preservation of the reading frame, which, leading to a change in the key positions of protein subunits, affect the ability of laminin α3, β3, and γ2 to assemble into a trimeric molecule, its secondary structure, and its ability to form intracellular anchor fibrils (Kiritsi et al., 2011).

A special phenotype, laryngo-onycho-cutaneous syndrome (LOC syndrome), manifests pathogenic variants that form a stop codon in exon 39, specific for the alpha-3 subunit of the LAMA3 gene, where three causative variants have been described so far: p.V51fs; p.Gln157Ter; p.Trp16Ter (Wang et al., 2022). Recently, C. Prodinger et al. (2021) reported three new mutations in the LAMA3 gene outside of exon 39.

Type XVII collagen protein is a homotrimer consisting of three identical subunits, is a transmembrane protein and the main structural component of PD, has both intracellular and extracellular domains. Type XVII collagen acts as a cell surface receptor for extracellular matrix proteins (van den Bergh, Giudice, 2003). The extracellular domain of type XVII collagen is associated with laminin-332; in this regard, it takes part in the creation of anchor filaments, can control cell motility, determines the spatial orientation of laminin-332 and its location in the collagen-IV-containing lamina BM (Tong, Xu, 2004).

This protein also regulates the differentiation of ameloblasts, epithelial cells involved in the formation of tooth enamel (Asaka et al., 2009). Enamel defects, ranging from punctate to generalized hypoplasia, occur in all subtypes of JEB, arising from impaired adhesion of the odontogenic epithelium from which ameloblasts originate (Wright et al., 2015).

Also, type XVII collagen plays a central role in regulating the proliferation of the interfollicular epidermis, participating in the maintenance of hair follicle stem cells, where it controls their aging program, which may explain the irreversible hair loss in people with type XVII collagen deficiency (Matsumura et al., 2016).

Pathogenic variants in the COL17A1 gene usually result in moderate JEB (Pasmooij et al., 2004), although a few fatal cases have been described with the presence of pathogenic COL17A1 variants (Murrell et al., 2007). According to D. Kiritsi et al. (2011) 69 % of the COL17A1 gene variants were nonsense variants, insertions or deletions, 19 % were missense variants, and 12 % were splice site variants. Pathogenic variants leading to exon skipping in the COL17A1 gene have a mitigating effect on the phenotype, allowing the production of a sufficiently functional protein (Condrat et al., 2019).

In some cases, nonsense mutations can cause mild manifestations of moderate generalized JEB due to alternative splicing mechanisms. It was shown that in patients with a homozygous nonsense mutation p.R795X in exon 33, COL17A1 mRNA is formed as a result of alternative splicing, which allows the production of a small amount of type XVII collagen.

Integrins are heterodimeric transmembrane receptors consisting of α- and β-subunits that form a functional receptor (Masunaga et al., 2017). In the epidermis, α3β1, α6β4, and α2β1 integrins are the most abundant. The α6β4 integrin binds to laminin-332 and to keratin filaments within the cell, which allows it to coordinate the cellular response and regulate adhesion, migration, and proliferation of keratinocytes. The α6β4 integrin is also involved in the formation of HD integrity and stability and interacts with type XVII collagen, plectin, and dystonin (Has, Nyström, 2015). The group of β1-integrins is associated mainly with the basal surface of keratinocytes and is involved in the formation of focal contacts. The α3β1 integrin is found both on the basal and lateral surfaces of basal keratinocytes, where it can participate in intercellular contacts.

The ITGA6 gene encodes the α6 subunit, the ITGB4 gene encodes the β4 subunit of the α6β4 integrin. Pathogenic variants in these genes, leading to premature termination of translation, form a severe phenotype that can be fatal in the neonatal period. Most of the mutations are in the ITGB4 gene; splicing site variants, small deletions and insertions, amino acid substitutions that lead to a rare subtype, JEB with pyloric atresia, have been described (Masunaga et al., 2017). Studies of genotype and phenotype correlations indicate that variants located in the extracellular domain of ITGB4 are usually associated with a more severe phenotype compared to those located in the cytoplasmic tail (Mariath et al., 2021). In the ITGA6 gene, single variants with loss of function in patients from consanguineous families are described, which are clinically manifested by early manifestation and often with a fatal outcome (Schumann et al., 2013; Masunaga et al., 2017).

The ITGA3 gene encodes the α3 integrin subunit, which is associated with the β1 subunit and forms the α3β1 integrin involved in interactions with extracellular matrix proteins, including laminins. The α3 integrin subunit is expressed in basal keratinocytes, podocytes, tubular epithelial cells, alveolar epithelial cells, and many other tissues (Bardhan et al., 2020).

Several cases of JEB with interstitial lung disease and renal abnormalities have been reported, associated with pathogenic variants in the ITGA3 gene, the expression of which in different tissues explains the multiple organ damage observed in patients. In addition, the relationship between the α3 integrin subunit and the cell membrane is complex, including posttranslational modifications, cleavage, heterodimerization with the β1 integrin subunit, and association with CD151. Amino acid substitutions can interfere with these events and act as null mutations, leading to severe disease (Has et al., 2012); variants that express a residual, truncated, or dysfunctional protein may result in a milder phenotype and improved survival (Liu et al., 2021).

Dystrophic EB (DEB) is divided into two main groups: dominant DEB (DDEB) and recessive DEB (RDEB). Clinical diversity includes 11 subtypes, with all subtypes having cutaneous and extracutaneous manifestations of varying severity. In general, RDEB is more severe than DDEB, ranging from severe skin manifestations with scarring and fibrosis, secondary complications, extracutaneous manifestations, and a high risk of squamous cell carcinoma, to mild skin fragility on the extremities or only nail dystrophy. However, there is a significant phenotypic overlap between AD and AR subtypes, which often makes it clinically difficult to establish the type of inheritance of DEB in a patient, especially if the proband is the only patient in the family.

DEB develops as a result of mutations in only one gene, the COL7A1 gene, which encodes type VII collagen, the main protein of anchor fibrils that provide BM attachment to the underlying dermis. Pathogenic variants in the COL7A1 gene lead to a disruption in the production and molecular structure of collagen, causing splitting of the upper layers of the dermis and destruction of anchor fibrils. The nature and location of pathogenic variants are important determinants of the phenotype (Hovnanian et al., 1997), which is determined by the expression and residual function of collagen VII (Mariath et al., 2020).

Type VII collagen is a non-fibrillar collagen synthesized by both epidermal keratinocytes and dermal fibroblasts and is localized in the BM zone below the epithelial layers, representing a homotrimer consisting of three identical α1 polypeptide chains (Uitto et al., 1992). Each α1 polypeptide chain contains a central collagen triple helix domain and terminal non-collagen NC-1 and NC-2 domains (Chung, Uitto, 2010). The triple helical domain consists of a repeating Gly-X-Y sequence interrupted by non-collagenous regions, the largest of which consists of 39 amino acid residues and is known as the “hinge” region.

The NC-1 domain mediates the attachment of anchor fibrils to the basement membrane and islets of collagen IV in the dermis (Bruckner-Tuderman et al., 2013). The NC-2 domain contains conserved cysteines involved in the formation of disulfide bonds, which provide a link between type VII collagen homotrimers. In addition, loops formed by anchor fibrils in the papillary dermis capture and mechanically hold interstitial collagen fibers, which are mainly represented by collagen types I, III and V.

Also, type VII collagen promotes the migration of keratinocytes, which is one of the stages of wound healing, providing their re-epithelialization (Woodley et al., 2008). It has been shown that in DEB the size or number of anchor fibrils is reduced, or they are absent (Uitto, Christiano, 1992), which determines the main mechanism and severity of the development of clinical manifestations. Impaired function of type VII collagen leads to deep skin defects, scarring of the mucous membranes, the formation of milia and fibrosis.

Hundreds of mutations in the COL7A1 gene associated with DEB are known (Sawamura et al., 2005; Has et al., 2020a). Thus, most cases of DDEB are the result of dominantnegative mutations. Approximately 75 % of DDEB patients have glycine substitution variants in the Gly-X-Y triple helical domain, especially in exons 73, 74, and 75 (Varki et al., 2007). At this hotspot, glycine residue substitutions can lead to greater protein destabilization than glycine residue substitutions within a long, continuous collagen segment, and variants near the hinge region cause protein misfolding and accumulation within cells (Chen et al., 2001). It is also suggested that exon 73 may encode amino acid residues important for the ability of type VII collagen to provide keratinocyte motility (Woodley et al., 2008).

Glycine as well as other amino acid substitutions and splicing variants outside the Gly-X-Y region are also found in DDEB, and intrafamilial phenotypic variability suggests that other factors may influence cell resistance to friction (Koss- Harnes et al., 2002).

Severe generalized RDEB usually results from the absence of a COL7A1 gene product resulting in null genetic variants on both alleles, about 30 % of which are nonsense stop codon or splicing variants resulting in large deletions, determining disease severity (van den Akker et al., 2011). Many patients with moderate RDEB are compound heterozygous for a premature stop codon and glycine substitution in the collagen domain, another missense variant or variants that disrupt splicing, resulting in destabilization of the triple helix or conformational changes in the protein that affect its functionality (Pânzaru et al., 2022).

This variety of combinations of genetic variants explains the wide range of clinical manifestations. So, for example, p.Gly2049Glu and p.Arg2063Trp variants, adjacent to the “hinge” region, reduce the ability to maintain fibroblast adhesion and lead to a significantly reduced ability to support keratinocyte migration, which slows down the healing of erosions in RDEB patients (Varki et al., 2007). Milder forms of RDEB are often caused by a combination of splicing and missense variants. Glycine substitutions may also occur in RDEB.

Kindler syndrome (KS) is a rare type of EB characterized by skin fragility and acral blistering from birth, development of skin atrophy, photosensitivity, poikiloderma, diffuse palmoplantar hyperkeratosis, and pseudosyndactyly (Lai-Cheong, McGrath, 2022). Morphologically, KS differs from other types of EB in that blistering is variable and can occur at different levels of the dermal-epidermal junction. KS develops as a result of pathogenic variants in the FERMT1 gene. The disease is inherited according to the AR type.

The FERMT1 gene encodes the Kindlin-1 protein, which is a multidomain focal adhesion protein. Kindlin-1 is involved in the connection between the actin cytoskeleton and the extracellular matrix through focal adhesion, as well as in integrin-associated signaling pathways (Has et al., 2011). The absence of Kindlin-1 leads to disorganization of keratinocytes as a result of incorrect integrin-mediated cell adhesion and migration (Rognoni et al., 2016). More than 90 pathogenic loss-of-function variants have been registered in the FERMT1 gene, including: missense, nonsense, and splicing variants; insertions; and Alu-mediated gene rearrangements that result in the absence of the Kindlin-1 protein or the production of a non-functional protein (Lai-Cheong, McGrath, 2022). Environmental factors play an important role in the phenotypic diversity of KS and determine the severity. X. Zhang et al. suggested that homologue 1 of the fermitin family is important for the suppression of UV-induced inflammation and DNA repair (Zhang et al., 2017).

## Conclusion

The multisystem manifestations of EB and the involvement of a significant number of proteins that provide mechanical stability of the skin in the pathogenesis are due to its genetic heterogeneity. Pathogenic variants in the genes encoding proteins of the epidermal and dermal anchoring complexes, as well as signal proteins that determine the integrity of the skin, lead to their structural and functional defects. EB is characterized by pronounced clinical variability and, at the same time, similar manifestations in different genotypes. Research and accumulation of the data of the natural history of disease and the genotype-phenotype correlations contribute to understanding the EB pathogenesis and determine the development of approaches for symptomatic and etiopathogenetic, in particular, gene therapy.

## Conflict of interest

The authors declare no conflict of interest.
